# Recovery of Natural Pyrazines and Alcohols from Fusel Oils Using an Innovative Extraction Installation

**DOI:** 10.3390/molecules30143028

**Published:** 2025-07-18

**Authors:** Waldemar Studziński, Michał Podczarski, Justyna Piechota, Marzena Buziak, Myroslava Yakovenko, Yurii Khokha

**Affiliations:** 1 Department of Food Analysis and Environmental Protection, Faculty of Chemical Technology and Engineering, Bydgoszcz University of Science and Technology, Seminaryjna 3, 85-326 Bydgoszcz, Poland; 2 Centre for Academic Entrepreneurship and Technology Transfer, Nicolaus Copernicus University in Toruń, Jurija Gagarina 7, 87-100 Toruń, Poland; mpodczarski@umk.pl; 3 Alpinus Chemia Sp. z o.o., Garbary 5D, 86-050 Solec Kujawski, Poland; jpiechota@alpinuschemia.com (J.P.); mbuziak@alpinuschemia.com (M.B.); ykhoha@alpinuschemia.com (Y.K.); 4 Institute of Geology and Geochemistry of Combustible Minerals, Naukova 3a, 79060 Lviv, Ukraine; myroslavakoshil@ukr.net

**Keywords:** fusel oils, waste management, recovery, alkylpyrazines, alcohols

## Abstract

The production of spirits generates significant amounts of waste in the form of fusel oils-previously treated mainly as an environmental problem. This paper presents an innovative installation designed to recover valuable components from this difficult waste. The key achievement is the effective separation and recovery of pyrazine derivatives-natural aromatic compounds with high utility value in the food, cosmetics and pharmaceutical industries. The designed system allows for the recovery of as much as 98% of pyrazines and isoamyl alcohol and isobutanol fractions with a purity above 96%, which is a significant advance compared to previous disposal methods. The installation was designed to be consistent with the idea of a circular economy, maximizing the use of by-products and minimizing losses. The results of the work indicate that fusel oils, previously perceived as waste, can become a source of valuable secondary raw materials, and the presented solution opens up new possibilities for the sustainable development of the alcohol industry.

## 1. Introduction

Problems related to waste management are currently one of the most important issues in modern chemical technology. Due to the high costs of disposal and the current waste management model, which emphasizes recovery, research is being conducted on finding methods for separating substances characterized by a high separation coefficient, at the lowest possible cost. This is particularly important when valuable components can be extracted from the waste [1]. Currently, separation techniques in environmental protection and management are based on many unit operationsfor the separation of multicomponent and/or multiphase systems. Therefore, when choosing a method for handling a given waste, its chemical composition, properties, and the process in which it was created should be taken into account [2,3]. One of the branches of industry in which waste is generated is the spirits industry. One of the more interesting wastes generated by this industry is fusel oils (FO). Fusel oils are a mixture of alcoholic fermentation by-products collected in the final stages of distillation of mash (malt, potato, corn, or molasses), during the production of raw spirit, at the stage of its final refining [4,5]. Fusel oils, being fermentation waste, consist mainly of fermentation by-products such as *n*-propanol, *n*-butanol, isobutanol, *n*-pentanol, iso-amyl alcohol, and esters of lower fatty acids. However, depending on the fermentation substrate, yeast strain, fermentation temperature, pH, and others, the composition of fusel oils can vary significantly [6,7]. Fusel oils are characterized by a very intense, distinctive odor. Several of their components are odorous compounds, which makes them a very desirable raw material of natural origin used in the cosmetic, pharmaceutical, and food industries-primarily for the production of fragrance compositions and flavorings. An important component of fusel oils with aromatic properties is the group of compounds known as pyrazines. Alkylpyrazines are of particular importance for the aromatic industry. The content of pyrazines in fusel oils can reach up to 2.5% by mass [8,9].

Fusel oils are waste from which valuable components can be recovered, which contributes to waste minimization and maximization of raw material utilization [6]. Natural chemical substances are extremely valuable and desirable on the market due to their unique properties, as well as the growing interest of consumers in ecological and sustainable products. Compared to their synthetic counterparts, these substances often exhibit greater bioavailability, lower toxicity, and better better tolerance by the human body [10]. Additionally, natural products are more in line with “clean label” trends, which emphasize minimizing the use of artificial additives in food products, cosmetics, and pharmaceuticals. In the cosmetics, pharmaceutical, and food industries, these substances are used not only for their scents and flavors but also for their potential health properties, such as antioxidant, antibacterial, or antiviral effects. Essential oils, aromas, and extracts from natural raw materials —including fusel oils— play a key role in creating unique products that stand out in the market. For this reason, the commercialization possibilities of natural chemicals is increasingly appreciated, and their market is growing at a rapidly. This, in turn, is driving research on the optimization of their extraction and processing methods [10,11,12,13]. In particular, alkylpyrazines, due to their unique fragrance profiles, are becoming an increasingly sought-after raw material in the production of luxury perfumes, food flavors, and high-quality cosmetics. In the context of sustainable development and the pursuit of waste minimization, the reuse of such natural compounds from industrial waste as fusel oils is not only economically beneficial but also aligns with the principles of a circular economy—an approach gaining importance in modern chemistry and production technology. Current methods of utilization of fusel oils mainly include their disposal by incineration or use as low-quality feed additives, which does not fully exploit their potential as high-value raw materials [8,14]. There is a lack of a comprehensive approach to the full processing of these wastes, which contradicts the zero-waste and circular economy concepts, both of which emphasize the maximum utilization of raw materials and the minimization of waste.

Taking this information into account, the authors have designed an innovative, comprehensive fusel oil management technology that combines alcohol fraction recovery with the selective sorption and desorption of pyrazines using resin. While both resin sorption and fractional distillation are well-documented techniques in the literature, the key added value of this work lies not in each technique individually, but in their integrated application to an exceptionally complex waste matrix such as fusel oil.

## 2. Results and Discussion

### 2.1. Determination of the Chemical Composition of Fusel Oil

In the first stage of the study, an analysis was carried out to determine the chemical composition of the fusel oil used for the study. Fusel oil is a brown, oily, multicomponent mixture of organic substances that is formed as a by-product of the rectification process of raw alcohol at the second stage of obtaining pure ethyl alcohol. The main components of crude fusel oil are alcohols (isobutanol, isoamyl alcohol), water, and a mixture of alkylpyrazines (Table 1).

**Figure 1:**
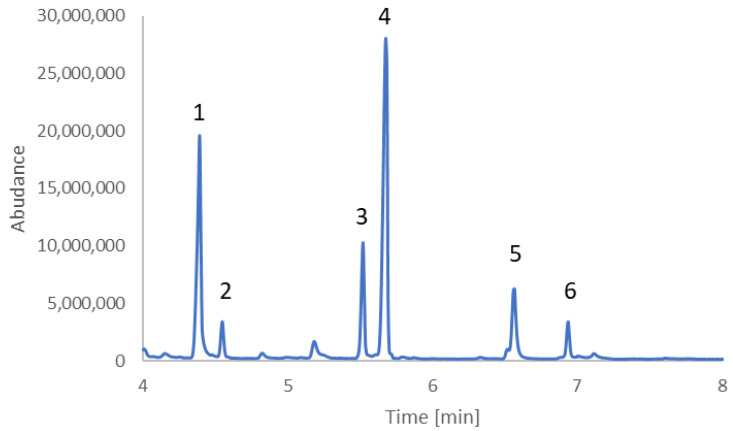
Chormatogram of alkylpyrazines present in fusel oil: (1) 2,3-dimethylpyrazine; (2) 2,5-dimethylpyrazine; (3) 2-methyl-3-ethylpyrazine; (4) 2,3,5-trimethylpyrazine; (5) 2,3-dimethyl-5-ethylpyrazine; (6) 2,3,5,6-tetramethylpyrazine.

Based on GC-MS analyses, it was found that fusel oil contains pyrazines such as: 2,3 dimethylpyrazine (2,3DMP), 2,5-dimethylpyrazine (2,5DMP), 2-methyl-3-ethylpyrazine (2M3EP), 2,3,5-trimethylpyrazine (2,3,5TMP), 2,3-dimethyl-5-ethylpyrazine (2,3DM5EP), 2,3,5,6-tetramethylpyrazine (2,3,5,6TMP) (Figure 1). The composition of alkylpyrazines in fusel oils is consistent with Van der Schaft [5] and Massa et al. [15].

**Figure 2:**
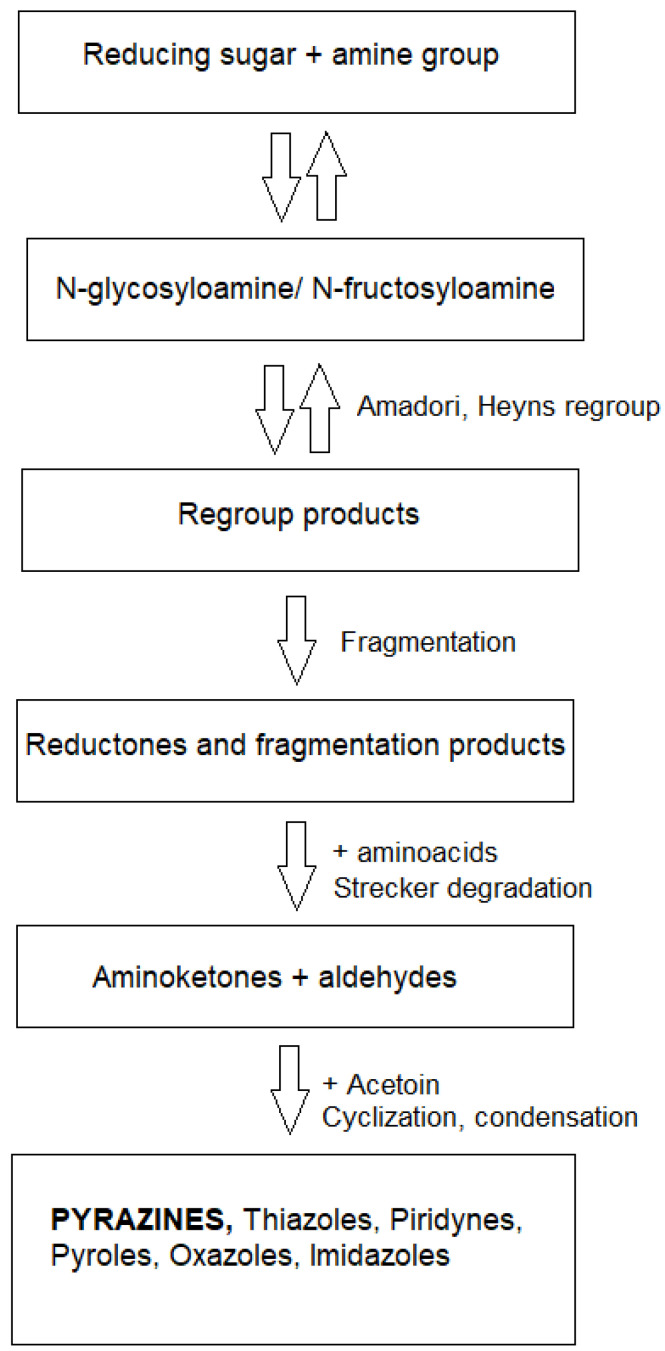
Schematic diagram of the processes occurring during the Maillard reaction.

The alkylpyrazine fraction in fusel oil is formed during the heating of the wort. It has been experimentally proven that pyrazines mainly originate from the Maillard reaction occurring during the rectification process. At temperatures above 70 °C, reducing sugars and amino acids can react and form a group of compounds called Maillard reaction products (Figure 2), which are coloring and have a strong taste [16].

Based on the conducted identification of alkylpyrazines, standards were purchased and it was decided to develop a quantitative method for determining pyrazines. For this purpose, calibration curves were prepared on GC-FID. It was found that the GC-FID method is linear and precise. The RSD of the analytes determined by the GC-FID method ranged from 2.7 to 5.9%. The GC-FID method allows for the determination of pyrazines at the level of μg L^−1^. The results are presented in Table 2.

### 2.2. Recovery of Valuable Components from Fusel Oils

Currently, most of the fusel oil waste is thermally processed, but scientists are working on alternative methods of waste management. Biotechnological utilisation may be an alternative. Treatment of fusel oil with enzymes, e.g., lipase, may lead to an esterification reaction, which may result in the formation of ethyl oleate [17]. Nemestóthy et al., in turn, carried out enzymatic esterification of oleic acid and isoamyl alcohol. The authors believe that the synthesis of isoamyl oleate by lipase is cost-effective [18]. Massa et al. carried out the treatment of FO in pressurized water, obtaining a solution resulting in antifungal activity compared to the reaction mixture (FO). In addition, the treatment of FO in pressurized water may promote its impregnation in wood, because after the reaction, compounds with smaller molecules are formed, which may result in a higher retention rate and, consequently, a greater ability to diffuse between wood layers [15]. In turn, other scientists use fusel oil as an additive to the fuel mixture. Çiftçi et al. studied the fuel mixture containing diesel oil, biodiesel and fusel oil on the operation of a 3-cylinder Lombardini LDW 1003 diesel engine. It was shown that the addition of 10% fusel oil reduces NOx and hydrocarbon (HC) emissions [19]. Simsek et al. showed that with the increase in the amount of fusel oil in the mixture, the emissions of nitrogen oxide (NOx), carbon monoxide (CO) and hydrocarbons (HC) decrease [20,21]. Alenezi et al. showed that even the addition of 30% FO does not deteriorate the fuel properties [22]. The above-mentioned methods of fusel oil management lead to the loss of valuable components.

**Figure 3:**
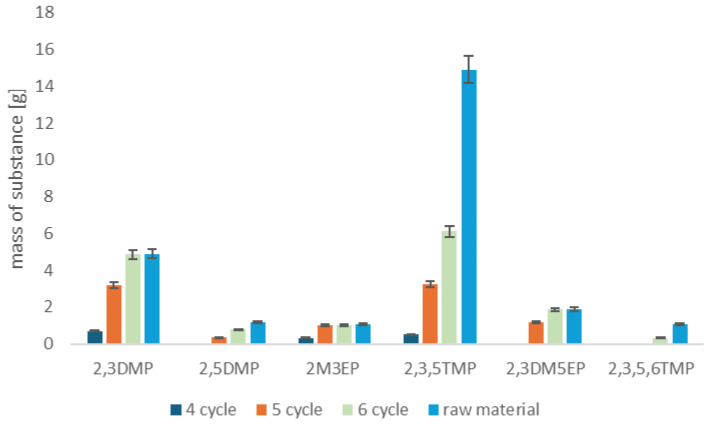
Recovery of alkylpyrazines from 1 L of fusel oil.

Based on the fusel oil analysis, it was found that the valuable components suitable for recovery are the pyrazine fraction, which constitutes 2.2% of the fusel oil content, and the alcohol fraction (76%). Hence, the authors attempted to develop a comprehensive technology for the recovery of components and the management of fusel oils. Therefore, the next goal of the research was to develop a method for the extraction of pyrazines from fusel oil and the separation of alcohols. First, a technology for extracting pyrazines on resin-packed columns was created and the process was carried out in accordance with the description of the installation for the recovery of pyrazines from fusel oils. After sorption on resin-packed beds, the pyrazine fraction was desorbed using methanol. The concentrate was then directed to a rectification column, where it was separated into methanol, a pyrazine fraction and a fraction containing medium and low volatility compounds. The pyrazine fraction was characterized by high purity of 98%. Depending on the demand for individual pyrazines or their mixture, the process can be repeated and individual pyrazines can be collected in separate fractions. The purified methanol was recycled to the desorption process. 1L of fusel oil was used in the experiment, which was passed through the installation 1–6 times in recirculation mode. In this way, the authors wanted to obtain the best possible extraction efficiency of pyrazine derivatives and to answer whether the amount of fusel oil recirculation affects the amount of sorbed pyrazines. Based on the obtained results, it can be stated that the amount of fusel oil recirculation through the installations affects the efficiency of pyrazine sorption. The first 4 cycles did not give satisfactory results regarding the recovery of alkylpyrazines (Supplementary Materials Figure S1a). The best results were achieved after the 6th cycle of fusel oil recirculation. After the 6th sorption cycle, the recovery of alkylpyrazines from fusel oil was 98% (Figure S1b). Figure 3 shows the amount of individual alkylpyrazines recovered from 1L of fusel oil.

The sorption of pyrazines and other aromatic compounds (PCs) on resins can be based on various physicochemical interactions—including van der Waals, hydrogen bonds, electrostatic bonds and π-π— the strength and nature of which depend on a number of factors. Determinants of the sorption process include the chemical structure of the resin (porosity, polarity, presence of functional groups), pH, temperature and impurity concentration, presence of interfering substances (carbohydrates, salts, oils), composition of the waste matrix— which can affect competitive adsorption and selectivity. The complexity of interactions in industrial systems (e.g., olive mill effluents or fermentation residues) means that resin selection and operating parameters must be tailored individually. Also important is the choice of desorption solvents selected for selective recovery of desired components. Importantly, systems such as ours based on continuous column operation may require an additional waste matrix clarification step to avoid bed clogging [23].

After extraction of pyrazines with methanol solution, the resin is regenerated with hydrochloric acid solution, water and methanol. The authors carried out 10 regenerations of the resin bed and did not observe any variation in the efficiency of pyrazine sorption on the resin. The results of pyrazine recovery on the resin, depending on the number of regenerations, are presented in the graph Figure S2. During the sorption process of pyrazines on resin beds, a residue containing water and alcohols was also obtained, which was discharged from the installation. The literature describes the recovery of alkylpyrazines from fusel oils derived from beet molasses using multiple distillation and rectification stages. In this way, more than 80% of alkylpyrazines can be economically isolated [5]. Pasquet et al. carried out the recovery of phenolic compounds from brewery liquid residues using the ultrafiltration process and adsorption on an ion exchange resin. Among the resins tested, the SCAV4 base resin with the OH- counterion showed the highest sorption capacity and selective sorption of phenolic compounds. The authors recovered up to 70% of phenolic compounds [23]. The literature also describes many extraction methods for recovering other bioactive compounds (antioxidants, preservatives, dyes, thickeners) from agro-food waste, which predominantly use organic solvents. Recently, green extraction technologies such as microwave-assisted extraction (MAE), ultrasound-assisted extraction (UAE) and supercritical fluid extraction (SFE) have played an important role [24]. Additionally, the use of natural eutectic solvents for the recovery of bioactive compounds has become an alternative to conventional extraction, which uses organic solvents. Supercritical extraction (SFE), despite its many advantages, is associated with significant limitations, such as high investment and operating costs resulting from the need to work under high pressure and complex infrastructure. Carbon dioxide, as a non-polar solvent, has limited effectiveness against polar compounds, which requires the use of additional modifiers and complicates the process. SFE also requires precise optimization of parameters and dry plant material, and working under pressure increases the operational risk. In turn, extraction using eutectic solvents (DES and NaDES), although promising, encounters difficulties related to high viscosity, the influence of water content, stability, and toxicity of the systems, and the lack of a universal solvent for various compounds. In both cases, scaling up the processes to an industrial level remains a major technological and economic challenge [10,24,25]. Green techniques seem promising, but require further research and optimization of the process [10,25]. Taking into account the above information, the sorption of pyrazines on resin presented by the authors offers a number of practical advantages compared to alternative methods. These advantages include: high selectivity towards pyrazines despite the complex matrix, the possibility of regeneration and repeated use of the resin, lower operating costs, no need for high pressure or specialized equipment (as in the case of SFE), and milder process conditions, which reduce the risk of analyte degradation.

**Figure 4:**
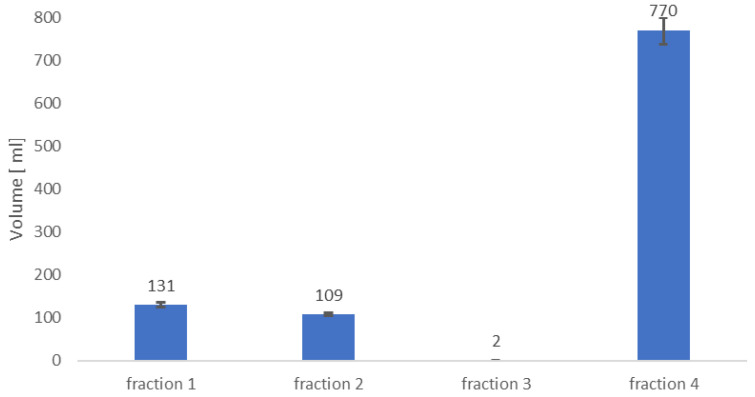
Content of individual fractions after fractional distillation of a water-alcohol mixture.

In the next stage of fusel oil development research, the authors used 906 mL of a water-alcohol mixture obtained in the previous process. Considering the properties of water and alcohols, it was decided to separate the mixture by fractional distillation. The fractions were collected in the following temperature ranges: fraction 1 (95–105 °C); fraction 2 (106–112 °C); fraction 3 (113–125 °C), fraction 4 (126–138 °C). In fraction 1, 1-propanol was identified in the distillate, which has a boiling point of 97.2 °C, and water. In fraction 3, the authors collected 1-butanol (boiling point 117 °C). The most valuable fractions in terms of chemical composition and the amount of distillate collected were fractions 2 and 4. In fraction 2, 108 mL of distillate was collected, which contained isobutanol (boiling point 108 °C). Fraction 4 was the most unique, the authors collected 770 mL of distillate, which contained isoamyl alcohol (boiling point 132.5 °C) (Figure 4). The main problem of fractional distillation and purification of alcohols is that alcohols can form an azeotropic mixture with water and often it is impossible to obtain anhydrous alcohol by means of a single distillation [26]. Taking this into account, the authors carefully monitored the temperature ranges of the obtained distillates to minimize this phenomenon. The obtained distillates were subjected to chromatographic analyses and analyses for water content in the sample. It is worth mentioning that the purity of alcohols in fractions 2 and 4 was above 96%. Details regarding the chemical composition of individual fractions are presented in Table S1. Additionally, fraction chromatograms are included in the Supplementary Materials (Figures S3–S6).

Due to the fact that fraction 1 is characterized by high hydration (water content above 90%), it can be directed to a sewage treatment plant and in this way, the water can be recirculated. Fractions 2 and 4 were characterized by high purity of the obtained isobutanol and isoamyl alcohol. Taking into account the above information, fractional distillation turned out to be a good way to recover isobutanol and isoamyl alcohol. Research conducted on the installation of Alpinus Chemia Sp. z o.o on an industrial scale gave similar results. Based on them, it was calculated that in this way, 99% of these alcohols can be recovered from the residues after the extraction of pyrazines from fusel oil. Recovery of alcohols is commonly carried out by other authors from post-fermentation raw materials. Montaño et al. obtained second-generation bioethanol from fermentation broths derived from wheat straw. Initially, the authors concentrated the fermentation broth using a distillation consisting of 15 stages, and then the actual batch distillation was carried out. The authors showed that a glycerol-to-ethanol ratio of 0.8:1 is sufficient to obtain fuel-grade ethanol. The purity of the ethanol fraction was above 95% [26]. Quiroz-Ramírez et al., after the fermentation process of lignocellulosic material, developed technologies for the purification and recovery of acetone, butanol, and ethanol using thermally coupled columns. Butanol is of particular importance, as it can be used as a biofuel [27].

The process of alcohol recovery and pyrazine sorption on resin was also carried out on an industrial scale. The technical and operational characteristics of the installation are presented in Table S2. The installation achieves very high recovery efficiency, 96–98% for pyrazines and 95–99% for isobutanol and isoamyl alcohol. The system is stable for at least 30 days of continuous operation, and the resin requires weekly cleaning due to acceptable contamination rates. Several potential challenges were identified during the operation. These include resin contamination, which is effectively limited by the use of pre-filtration and an optimized regeneration protocol. The installation is also rated by significant energy demand. The storage of end products requires special safeguards due to the flammability of alcohols. An additional challenge is the need to develop the sales market by gradually building a network of recipients. In terms of process control, automated switching of sorption columns was used, which allows for uninterrupted operation of the system. Resin breakthrough curves are monitored in real time, which allows for a quick response to changes in efficiency. The studies carried out show that from waste, which is currently mostly thermally neutralized, it is possible to recover components that could be used in various industries. Isoamyl alcohol is a raw material that is widely used in the production of flavorings. It can also be used to disinfect all surfaces from most microbes, viruses, and bacteria that may accumulate on everyday objects. It is also suitable as a diluent for various types of paints [28,29]. It is known that isobutanol is mainly used as a solvent in adhesives or surface coatings. It is also used in paints (inks for toners), cleaning agents, metalworking fluids, lubricants, and rolling oils. It can also be used in organic synthesis [30,31]. In turn, pyrazine derivatives, in addition to their fragrance function, shaping, and improving organoleptic properties, exhibit antioxidant activity. Recently, there have been more and more scientific reports on the biological and pharmacological properties of 2,3,5,6-tetramethylpyrazine, 2,5-dimethylpyrazine, and 2,3,5-trimethylpyrazine. Tetramethylpyrazine is an active ingredient of the herb *Ligiisticum wallichii*, used in Chinese medicine. Its action involves, among others, inhibiting platelet aggregation and alleviating neurological changes associated with spinal cord ischemia. This com-pound has been used in the treatment of cardiovascular and cerebrovascular diseases [32,33,34]. 2,3,5,6TMP has also neuroprotective, anti-inflammatory, and anticancer properties, which make it a promising therapeutic compound. 2,5-DMP affects the hormonal and lipid systems, acts on the central nervous system, and can play a supporting role in the therapy of reproductive and metabolic disorders. 2,3,5TMP, on the other hand, shows pheromone activity and potential as a stabilizer of co-crystals in pharmaceutical chemistry, and is also considered a sustainable by-product in industry. All of these compounds have beneficial pharmacological applications, which emphasizes their industrial importance [4,5,6,7,8,9,10,11,12,13,14,15,16,17,18,19,20,21,22,23,24,25,26,27,28,29,30,31,32].

**Figure 5:**
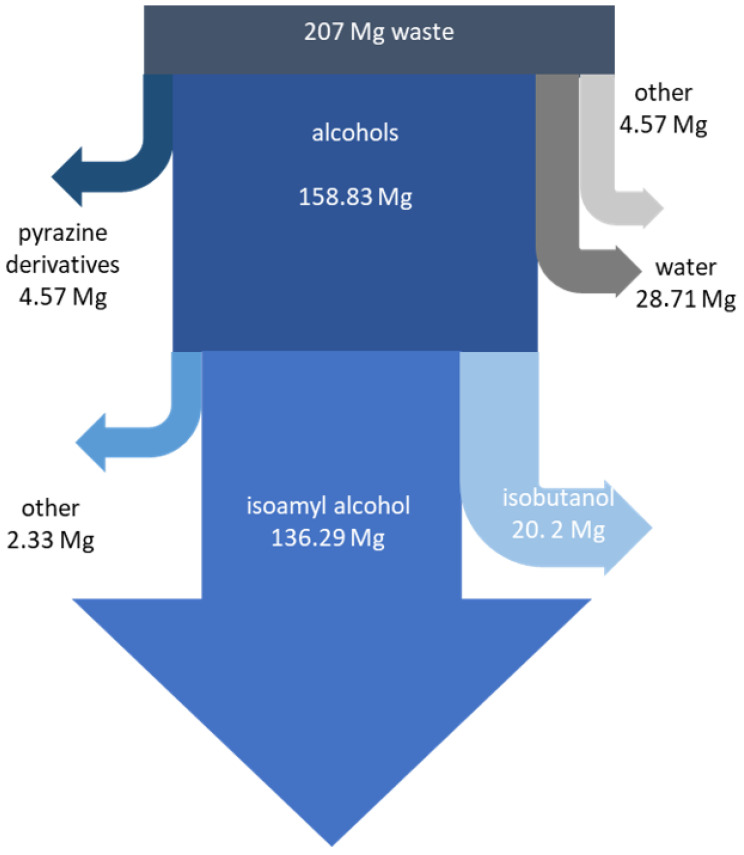
Recovery of individual chemicals from fusel oils per year.

In connection with the above, the authors carried out calculations of the recovery of substances from fusel oils from distilleries on an annual basis. In a local distillery, where the production capacity of spirit is 34.5 million L, about 207 Mg of fusel oils are produced annually. The cost of thermal waste disposal is EUR 410,400. The pyrazine derivative recovery installation allows for the recovery of 4.57 Mg of pyrazine derivatives (Figure 5). Additionally, fractional distillation allows for the recovery of high purity 136.29 Mg of isoamyl alcohol and 20.2 Mg of isobutanol. Additionally, about 30 Mg of the water fraction with the addition of 1-propanol can be directed to a sewage treatment plant and reduce the waste mass that needs to be disposed of. Finally, after comprehensive management of individual valuable components present in fusel oil, about 5 Mg of final waste remains. The characteristics of the residue include mainly high molecular weight organic compounds (55%). In the residual waste are also inorganic salts from the fermentation process (30%) and fine resin residues and other process residues (15%). In accordance with the concept of closed circulation and zero waste, the authors recommend thermally processing the residual waste with energy recovery, especially since previously, the entire mass of the waste was managed in this way. This translates into a significantreduction in waste mass and a reduction in the waste disposal cost to EUR 7600. Additionally, taking into account the prices of individual substances that can be recovered, e.g., 1 kg of isoamyl alcohol costs EUR 30.50; the price of 1 kg of isobutanol is EUR 11.73; 25 g of 2,3-dimethylpyrazine costs EUR 221.47; and the price of 25 g of 2,3,5-trimethylpyrazine is EUR 146.72.

The preliminary economic analysis was supplemented with available input data and financial results obtained from Alpinus Chemia (Table S3). Although the assumptions were based on available market and catalog data (e.g., wholesale chemical prices, labor costs), we also conducted a sensitivity analysis and Monte Carlo simulation (10,000 iterations), which showed an 89% probability of achieving a positive NPV under uncertainty in prices, productivity and operating costs. The purpose of this preliminary analysis was to estimate the economic potential of the process in screening terms-i.e., to determine whether the developed technology is within the limits of potential profitability and whether it warrants further development research. Thus, despite the lack of full data from real industrial applications, this analysis provides a valuable starting point for further economic verification of the process under real conditions. The authors are convinced that the recovery of the substance will be economically profitable.

## 3. Materials and Methods

### 3.1. Chemical Reagents

The following analytical standards of pyrazines were used in the studies: 2,3-dimethylpyrazine (2,3DMP) (98%), 2,5-dimethylpyrazine (2,5DMP) (99%), 2-methyl-3-ethylpyrazine (2M3EP) (98%), 2,3,5-trimethylpyrazine (2,3,5TMP) (99%), 2,3-dimethyl-5-ethylpyrazine (2,3DM5EP) (98%), 2,3,5,6-tetramethylpyrazine (99%) from Sigma-Aldrich. Methanol (99.9%) (Sigma-Aldrich, Saint Louis, MO, USA) was used as a solvent. Fusel oil from a distillery in south-western Poland was used for the studies.

Styren Post Crosslink resin with DVB (H103) with a particle size of 0.3–1.2 mm and a specific surface area of 1000–1100 m^2^/g was used to fill the sorption columns. The resins were purchased from Bengbu Dongli Chemical Co. (District, Bengbu, Anhui, China).

### 3.2. Characteristics of the Installation for Recovery of Pyrazines from Fusel Oils

**Figure 6:**
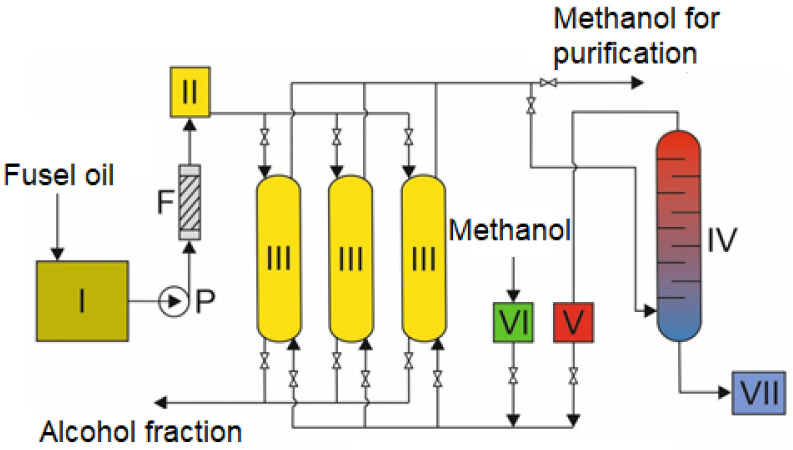
Schematic diagram extraction of derivatives of pyrazine from fusel oil: I—fusel oil tank; P—pump; F—filter eliminating solid particles; II—tanks for distributing raw materials in resin columns; III—resin columns, two working in sorption mode, one in regeneration mode; IV—rectification column for separating the desorbing agent and pyrazines; V—tank for pure desorbing agent; VI—tank for pure methanol; VII—container for pyrazines concentrate.

In this work, an innovative installation for the recovery of pyrazines from fusel oils in a closed circuit was designed and used. The installation diagram is shown in Figure 6.

According to the scheme, the fusel oil is transported to a container (I), from where it is pumped through a filter (F) to remove solid particles present in the raw material. The purified fusel oil is fed to a container (II) and then dosed onto columns with resin at a flow rate of 7–10 mL/min (III). Each column was filled with 60 g of H103 resin. Pyrazines present in the fusel oils are sorbed onto the resin. The remaining mixture that passes through the column is discharged from the installation. A desorption solution (methanol) is used to desorption pyrazines from the resin-filled columns, which is dosed from the container (V) to the columns. The solution with pyrazines is then directed to the rectification column (IV), where it is separated into two fractions: a pure desorption reagent (reused) and a pyrazines concentrate. This concentrate is collected in a container (VII), from where it is taken for further separation into narrow fractions or pure substances. After desorption, the resin columns are washed with pure methanol from container (VI). The contaminated methanol from the wash goes to a thin-film distiller, where it is purified, and then returned to the production cycle.

### 3.3. Fractional Distillation of a Water-Alcohol Mixture

The water-alcohol residue from the process of recovering pyrazines from fusel oil was subjected to fractional distillation in order to separate the components of the mixtures and check the possibility of their recovery. The mixture consisted of water, 1-propanol, 1-butanol, isoamyl alcohol, and ethyl alcohol. The water-alcohol mixture was subjected to distillation, collecting the distillates in receivers at different boiling points. Temperature ranges [°C] were distinguished: fraction 1 (95–105 °C); fraction 2 (106–112 °C); fraction 3 (113–125 °C), and fraction 4 (126–138 °C).

### 3.4. Chromatographic Analysis of Fusel Oils and Extracts Containing Pyrazines

A sample of fusel oils and extracts containing pyrazines was dissolved in methanol and then filtered through a PTFE filter with a diameter of 0.45 µm. The samples prepared in this way were analyzed using GC-FID (Agilent Technologies 7890B, Santa Clara, CA, USA) and GC-MS (Agilent Technologies 7890B coupled with an Agilent Technologies 5977B MSD detector, Santa Clara, CA, USA). Alcohol fractions were analyzed qualitatively using GC-MS HS-SPME. Alcohol samples were also analyzed quantitatively using GC-FID. Compounds were separated on an HP-5MS column (30 m × 0.25 mm × 0.25 µm), and helium was used as the carrier gas, flow rate of 1 mL/min. Oven temperature program: 100–10 °C/min–250-1 °C/min. Injection volume: 1 μL, split ratio 1:10. The detector and dispenser temperature were set to 260 °C.

### 3.5. Water Content Analysis

Water content in fusel oil determined by volumetric Karl Fischer titration using methrom’s OMNIS KF (Herisau, Switzerland).

## 4. Limitations and Future Perspective

Despite the promising results obtained in this study, several important limitations must be acknowledged, as they may affect the comprehensive evaluation of the efficiency and scalability of the proposed process. One of the main limitations is the use of fusel oils from a single source, which restricts the generalizability of the findings. Additionally, the experimental data were primarily derived from laboratory-scale studies and preliminary observations from an industrial-scale setup, necessitating further long-term validation.

The economic analysis presented was based on current market conditions, which are subject to change, and the long-term durability of the system has yet to be confirmed. Thus far, it has been demonstrated that pyrazine sorption efficiency remains stable after 10 regeneration cycles. However, to fully assess the long-term chemical and mechanical stability of the resin, a detailed physicochemical analysis of the used material—employing techniques such as FTIR, XPS, or SEM—would be advisable.

Moreover, comprehensive characterization of sorption kinetics, isotherms, and mass transfer limitations could provide a robust foundation for future process optimization. Industrial-scale trials also revealed several operational challenges, including the risk of resin contamination, the need for precise energy management, and the requirements for the safe storage of flammable products. In the context of continuous operation, implementing automatic column switching, real-time monitoring of breakthrough curves, and systematic quality control of both product and waste streams is of critical importance.

Future work should focus on expanding the research to include process validation using fusel oils from diverse sources, conducting long-term operational tests (e.g., over six months), evaluating alternative resins with enhanced selectivity, and exploring the application of microreactor technologies to intensify the process. Another key direction will be the integration of the developed technology within the biorefinery concept, enabling comprehensive valorization of agro-food waste

## 5. Conclusions

This article presents an alternative method for managing waste generated during spirit production. The research conducted has demonstrated that the technology developed by the authors enables the comprehensive utilization of fusel oil. Traditionally, fusel oil—a by-product of spirit distillation—has been subjected to thermal neutralization. However, the authors have shown that the implementation of a pyrazine recovery system combined with fractional distillation of the water–alcohol mixture can reduce the total waste mass and disposal costs by up to 97.5%.

As a result of pyrazine isolation, compounds such as 2,3DMP; 2,5DMP; 2M3EP; 2,3,5TMP; 2,3DM5EP; and 2,3,5,6TMP can be recovered, while distillation allows for the separation of pure fractions of isoamyl alcohol and isobutanol. The data presented in the article indicate that the recovery of alcohols and alkylpyrazines is both environmentally and economically viable.

This approach to waste utilization aligns with circular economy principles and offers a sustainable, value-added pathway for managing by-products from the spirits industry. According to the authors, the proposed solution is not only technically feasible but also economically justified.

## 6. Patents

This manuscript is based on Polish patent application no. P.450887.

## Figures and Tables

**Table 1:** Composition of fusel oils.

Chemical Composition	Percentage Content [%]	*m/z*
water	13.87	-
1-propanol	0.96	31 (100), 27 (12), 60 (10)
1-butanol	0.17	41 (70), 43 (52), 56 (100)
izobutanol	9.76	31 (52), 41 (65), 43 (100)
Isoamyl alcohol	65.84	42 (100), 55 (66), 70 (51)
2,3DMP	0.49	67 (100), 108 (70), 109 (20)
2,5DMP	0.12	40 (40), 42 (100), 108 (60)
2M3EP	0.11	67 (83), 94 (73), 121 (100), 122 (86)
2,3,5TMP	1.49	42 (100), 81 (33), 122 (50)
2,3DM5EP	0.19	42 (28), 135 (100), 136 (75)
2,3,5,6TMP	0.11	54 (100), 95 (15), 136 (64), 137 (15)
other compounds	6.9	-

**Table 2:** Selected parameters of the GC-FID method for determining pyrazines.

	LOD	LOQ	R^2^	RSD	Linear Range	Calibration Equation
	µg L^−1^			[%]	[mg/L]	
2,3DMP	1.4	4.2	0.9961	5.1	0.5–50	y = 1.247x + 0.023
2,5DMP	2.3	6.9	0.9777	5.3	0.5–50	y = 0.998x + 0.041
2M3EP	0.8	2.4	0.9962	5.6	0.5–50	y = 1.156x + 0.015
2,3,5TMP	0.47	1.41	0.9987	4.2	0.5–50	y = 1.289x + 0.008
2,3DM5EP	2.36	7.08	0.9381	5.9	0.5–50	y = 0.876x + 0.052
2,3,5,6TMP	0.26	0.78	0.9987	2.7	0.5–50	y = 1.334x + 0.012

Method Robustness: Systematic variation of analytical parameters (±5% temperature, ±4% flow rate, ±2% injection volume) showed no significant impact on quantitative results (RSD < 3%), confirming method reliability.

## Data Availability

The data presented in this study are available on request from the corresponding author.

## Supplementary Materials

The following supporting information can be downloaded at: https://www.mdpi.com/article/10.3390/molecules30143028/s1, Figure S1: (a) pyrazine sorption capacity in resin, (b) pyrazine recovery depending on the recirculation cycle; Figure S2: Pyrazine recovery depending on the regeneration cycle; Table S1: Detailed Characterization of Alcohol Fractions; Figure S3: chromatogram of fraction 1, major peak is 1 Propanol; Figure S4: chromatogram of fraction 2, major peak is Isobutanol; Figure S5: chromatogram of fraction 3, major peak is Isobutanol; Figure S6: chromatogram of fraction 4, major peak is Isoamyl alcohol; Table S2: Technical and operational characteristics of the installation; Table S3: The economic analysis.
