# Supplementary material for: Recovery of Natural Pyrazines and Alcohols from Fusel Oils Using an Innovative Extraction Installation
Source: Molecules. 2025 Jul 18;30(14):3028. doi: 10.3390/molecules30143028 (PMC12300360; doi:10.3390/molecules30143028)
Supplement: Supplementary file 1 [file molecules-30-03028-s001.zip › molecules-3681890-supplementary.pdf]

Recovery of natural pyrazines and alcohols from fusel oils using an innovative extraction installation

Waldemar Studziński [1*], Michał Podczarski [2], Justyna Piechota [3], Marzena Buziak [3], Myroslava Yakovenko [4] and Yurii Khokha [3]

[1] Department of Food Analysis and Environmental Protection, Faculty of Chemical Technology and Engineering, Bydgoszcz University of Science and Technology, Seminaryjna 3, 85-326, Bydgoszcz, Poland

[2] Centre for Academic Entrepreneurship and Technology Transfer, Nicolaus Copernicus University in Toruń, Jurija Gagarina 7, 87-100Toruń, Poland ; mpodczarski@umk.pl

[3] Alpinus Chemia Sp. z o.o., Garbary 5D, 86-050 Solec Kujawski , Poland; jpiechota@alpinuschemia.com (J.P.); mbuziak@alpinuschemia.com (M.B.); ykhoha@alpinuschemia.com (Y.K.)

[4] Institute of Geology and Geochemistry of Combustible Minerals, Naukova 3a, 79060, Lviv, Ukraine; myroslavakoshil@ukr.net

* Correspondence: waldemar.studzinski@pbs.edu.pl, tel.: +48 52 374 90 67, fax.: +48 52 374 90 05

a)

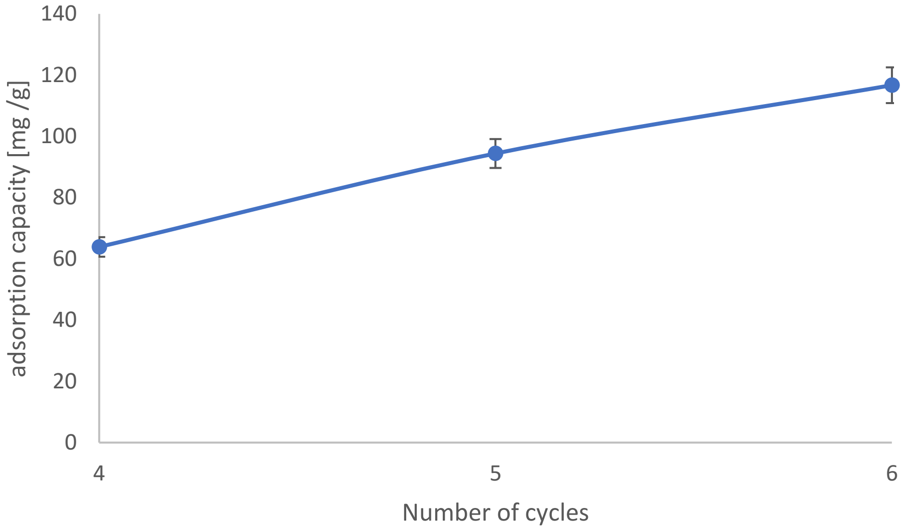

b)

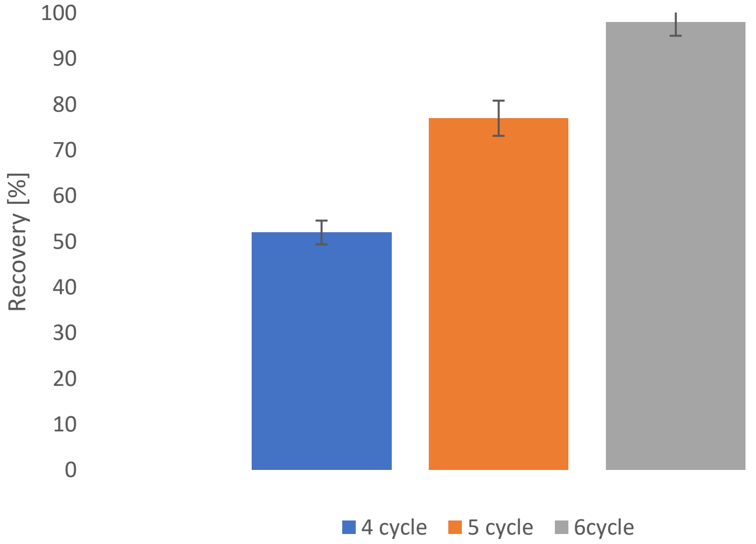

Figure S1. a) pyrazine sorption capacity in resin, b) pyrazine recovery depending on the recirculation cycle

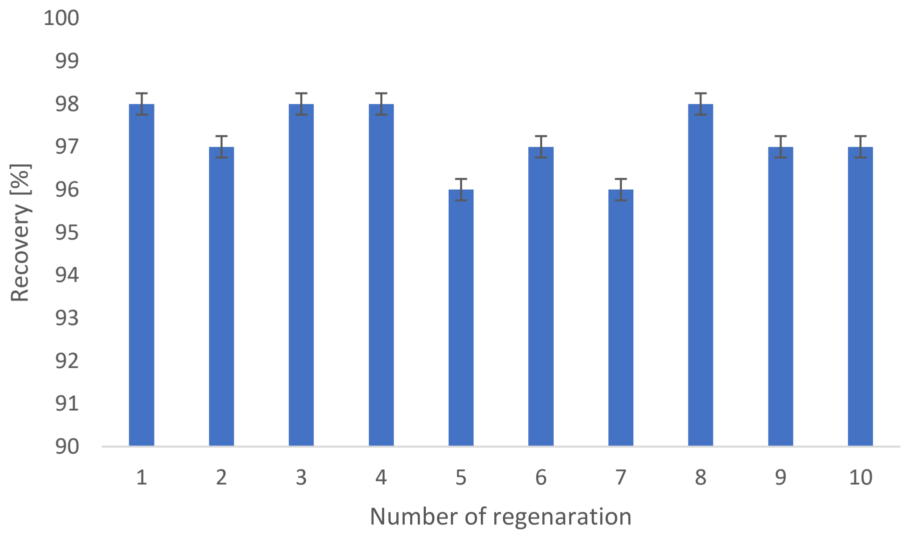

Figure S2. Pyrazine recovery depending on the regeneration cycle

Table S1. Detailed Characterization of Alcohol Fractions

**Fraction**	**Volume (mL)**	**Main Component**	**Purity [%]**	**Water Content [%]**	**Secondary Component(s)**
1	28	1-Propanol	8.5	91.2	Trace alcohols
2	108	Isobutanol	96.4	2.1	1-Propanol (1.5 %)
3	12	1-Butanol	94.8	3.2	Isobutanol (2.0 %)
4	770	Isoamyl alcohol (3-methyl-1-butanol)	96.8	1.8	Higher alcohols (1.4 %)

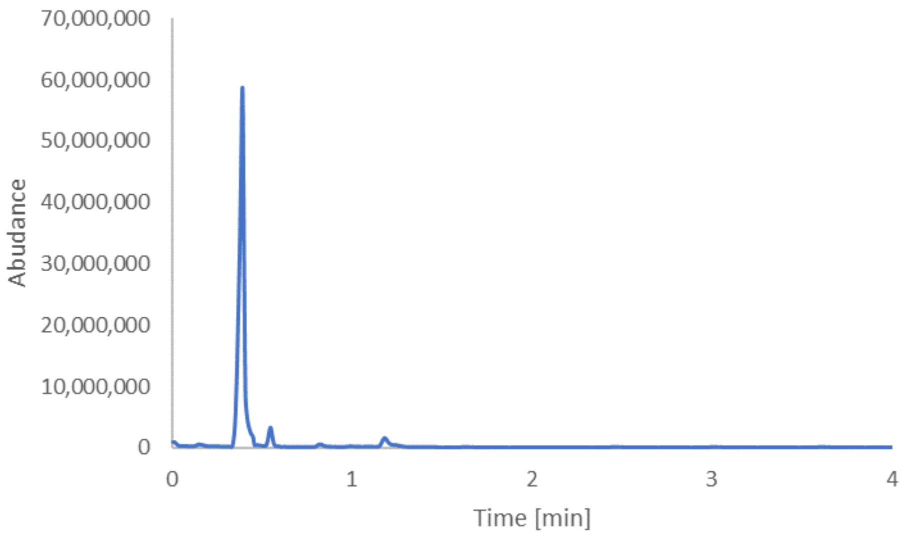

Figure S3. chromatogram of fraction 1, major peak is 1 Propanol

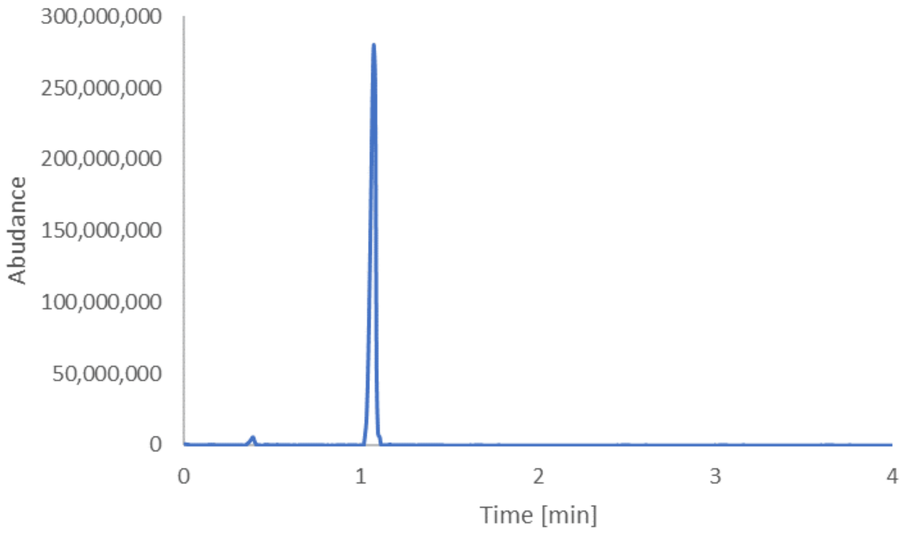

Figure S4. chromatogram of fraction 2, major peak is Isobutanol

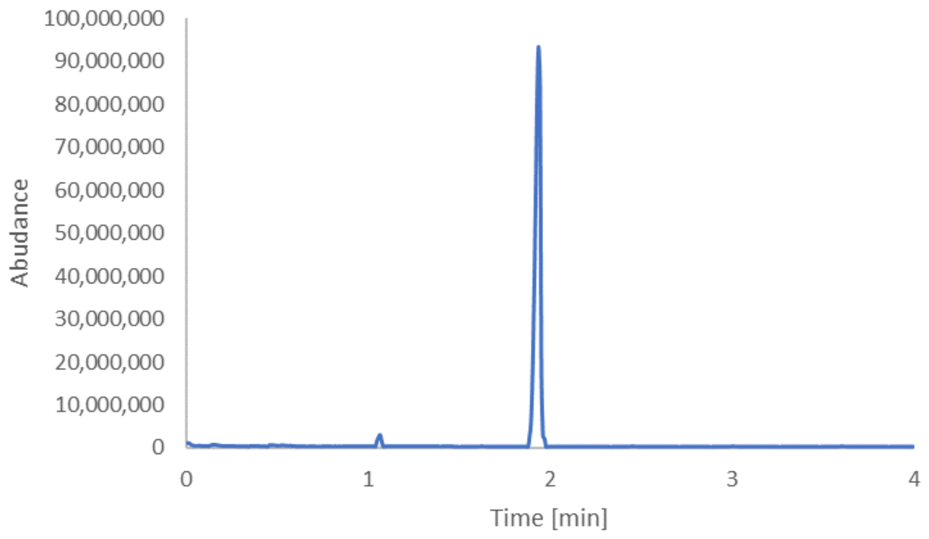

Figure S5. chromatogram of fraction 3, major peak is Isobutanol

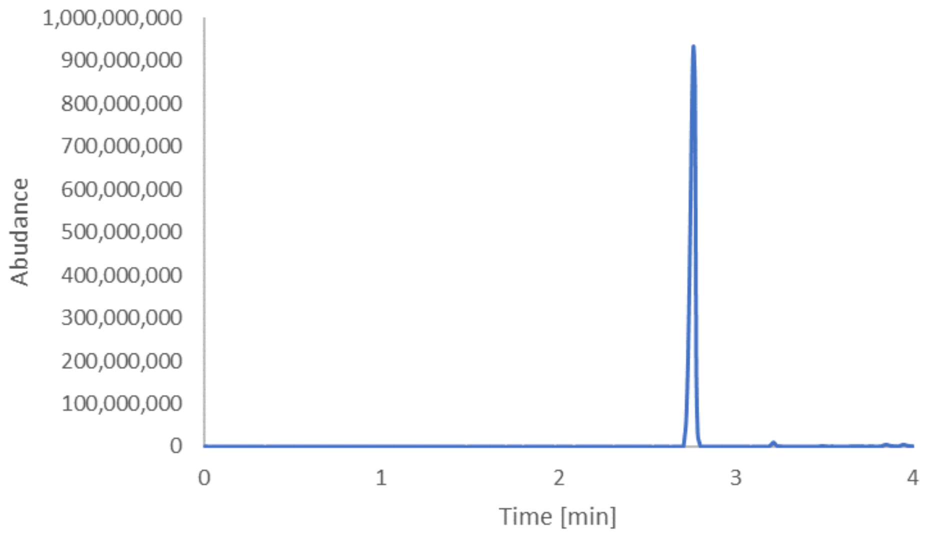

Figure S6. chromatogram of fraction 4, major peak is Isoamyl alcohol

Table S2. Technical and operational characteristics of the installation

Category	Parameter / Description
**Technical Specifications**	
Feed volume	100 liters of fusel oils
Number of columns	3 adsorption columns
Column packing	H103 resin (1000 g per column)
Flow rate	70–100 ml/min
**Performance & Operation**	
Pyrazine recovery	96–98%
Isobutanol and isoamyl alcohol recovery	95–99%
Continuous operation period	30 days without interruption

Resin cleaning frequency	Weekly, due to permissible contamination thresholds
**Operational Challenges**	
Resin contamination	Minimized via pre-filtration and optimized regeneration protocol
Energy demand	Balanced through heat integration and energy recovery
Product storage	Requires proper facilities due to alcohol flammability
Market development	Needs gradual market entry and customer base expansion
**Process Control**	
Automation	Automated switching between adsorption columns
Process monitoring	Real-time breakthrough curve monitoring
Quality control	Batch-by-batch product quality testing
Waste stream management	Waste stream monitoring to ensure compliance with environmental regulations

Table S3. The economic analysis

Category	Position	Value [EUR]
**Capital Expenditure (CAPEX)**	Resin columns and associated equipment	105,000
	Rectification system	75,000
	Control and monitoring systems	25,000
	Installation and commissioning	50,000
	**Total CAPEX**	**255,000**
**Operating Expenditure (OPEX) per annum**	Utilities (electricity, steam)	18,500
	Consumables (methanol, regeneration chemicals)	12,300
	Labor	25,000
	Maintenance and depreciation	11,500
	**Total OPEX**	**67,300**
	Isoamyl alcohol (136.29 Mg)	4,156,845

**Revenue Analysis (based on annual 207 Mg fusel oil processing)**	Isobutanol (20.2 Mg)	236,946
	Alkylpyrazines (4.57 Mg)	2,847,500*
	**Total revenue**	**7,241,291**
**Sensitivity Analysis**	Base case NPV (10 years, 8% discount)	52,400,000
	Pessimistic scenario (-30% prices)	36,700,000
	Optimistic scenario (+20% prices)	63,200,000
**Break-even analysis**	Required revenue level relative to the base case scenario	23%
**Risk Assessment: Monte Carlo simulation**	Probability of positive NPV (10,000 iterations) with price volatility (±25%), yield variations (±10%), and operational costs (±15%)	89%

*Conservative estimate based on average market prices with 30% discount for bulk sales
